# Empirical analysis of factors influencing student satisfaction with online learning systems during the COVID-19 pandemic in Thailand

**DOI:** 10.1016/j.heliyon.2022.e09183

**Published:** 2022-03-24

**Authors:** Piriyakorn Kornpitack, Sudaporn Sawmong

**Affiliations:** KMITL Business School (KBS), King Mongkut's Institute of Technology Ladkrabang (KMITL), Bangkok, Thailand

**Keywords:** Distance education, High school, Online education, Student satisfaction, Thailand

## Abstract

Starting in early 2020, Thailand's education system came to a grinding halt due to the global COVID-19 pandemic, which created a fervor-like effort to move from traditional classrooms to online education. However, the process has experienced significant troubles. Therefore, starting in June 2021, multiple-stage random sampling and simple random sampling were used to select a sample of 270 Thai high school students across nine Thai provinces. Using a network of Thai teachers, students were assisted with their questionnaire input using Google Form. LISREL 9.1 software was used to conduct the subsequent goodness-of-fit (GOF) assessment and the confirmatory factor analysis (CFA). A structural equation model (SEM) was used for the 53-item questionnaire, which contained eight latent variables, 18 observed variables, and ten hypotheses. Descriptive statistics were used to analyze the SEM's output and ten hypotheses. After that, it was calculated that the model's causal variables had a positive effect on SS, which had an R^2^ of 54%. The analysis also revealed that when ranked by total effect (TE) values, *performance expectancy* (PE = 0.43) was most significant, followed by *actual use* (AU = 0.30), *learner interaction* (LI = 0.18), and *behavioral intention* (BI = 0.12). Overall, hypotheses testing established three moderately strong correlations, four weak correlations, and three unsupported hypotheses. The novelty of our study is the growing concern of stakeholders for how online learning affects student satisfaction due to the deadly global COVID-19 pandemic. This study's research contribution is that it is unique in that it was conducted during the pandemic lockdown while students were participating in Thai Ministry of Education (MOE) online courses. This paper contributes to the online education domain by providing research directions and implications for future researchers. In conclusion, the study confirmed that the model adequately explained causal relationships between variables and presented direct and indirect significant impacts on online SS, promoting learners' better academic performance and knowledge acquisition.

## 1 Introduction

As recently as May 2021, UNESCO tragically revealed that due to the COVID-19 pandemic, 90% of the world's school-aged children had their education disrupted, with 26 countries experiencing a total shuttering of their traditional, classroom-based school systems (Human Rights Watch, 2021; UNESCO, 2021). Although Thailand did not ‘shutter’ its educational system totally, it did move a vast percentage of it online. In so doing, millions of students and teachers have been thrown into a vast experiment with little to no preparation for what has come. In this vast 'experiment,' student satisfaction for online learning and studying has become a growing concern. Therefore, it has become imperative that researchers heed this call and undertake research to identify and examine how online factors affect student satisfaction in the wake of the COVID-19 pandemic (Krouska et al., 2021).

The focus and shift to online education in Thailand have been swift and decisive but far from perfect or easy. Moreover, teachers are now confronted with even more monumental problems beyond the more straightforward issues of social distancing and wearing masks that the pandemic has brought with it. These include no home internet, lack of parental support, and chaos at home. Also, there is a lack of resources, irregular schedules, student workload in care for their families, food availability, and home and housing uncertainty (Human Rights Watch, 2021; Wangkiat, 2021).

Furthermore, student satisfaction has been a leading indicator of academic quality experiences, making it essential for determining a program's quality (Al-Rahmi et al., 2020). Before online learning, numerous studies existed concerning student satisfaction. However, studies have shifted from traditional classroom settings to the online world due to the pandemic, changing the traditional learning environment (Parahoo et al., 2015). Therefore, this raises the question of whether factors impacting student happiness differ in real-world and virtual settings, with more and more research is being conducted on the issues affecting student happiness online (Baber, 2020; Hebebci et al., 2020).

Singh and Thurman's (2019) reviewed the terms ‘*online education*’ and ‘*online learning*' and reviewed all publications that defined the word from 1988 to 2018. Moreover, they described '*online education*' as education that uses the Internet for teaching and learning in an online setting. Online learning not based on a student's physical or virtual location falls under this category. Teachers create synchronous or asynchronous instructional modules to boost learning and engagement, and the information is provided online.

However, studies concerning online learning student satisfaction are limited in Asian cultures compared to Western Culture (Parahoo et al., 2015). There are diverse cultural, economic, and technological landscapes, so doing similar investigations in developing nations like Thailand is important (Darawong and Widayati, 2021); Harsası and Sutawıjaya, 2018; Parahoo et al., 2015; Thongsri et al., 2019). This is particularly so, as, in August 2021, Thai student discontent over the quality and content of their online classes reached new heights, with the group ‘*Bad Students*’ instituting online class strikes and mock student suicides at major intersections in Bangkok (Wangkiat, 2021).

Therefore, student satisfaction is essential in distance learning evaluation as it is concerned with online learning quality and student performance (Harsası and Sutawıjaya, 2018). Furthermore, in a study from the US-based International Association for K–12 Online Learning (iNACOL), the association says a pillar for personalized learning is through online education (Friend et al., 2017). Reasons for this come from the new idea of ‘*voice and choice*' in which students can choose when, what, how, and where their learning occurs. Thus, educators act as facilitators in creating flexible learning environments and processes.

In Thailand, the Ministry of Education (MOE), even before the COVID 19 pandemic, had started significant initiatives in developing information and communication infrastructure (ICT) under the MOE's *Thailand ICT Framework* (ICT 2020) and a *Smart Thailand 2020* strategy (Ruenphongphun et al., 2021). Under these strategies, key components were outlined in which ICT access would be expanded through an ongoing ICT infrastructure development program, including greater mobile broadband penetration and increased Thai citizen information and digital literacy (Oranop, 2016). However, new leaders modified, improved, and renamed the *Digital Economy Master Plan* (DEMP) plans, under which five main programs were outlined. These include expanding and improving complex infrastructure, service and soft infrastructure, innovation and promotion, and finally, Thai knowledge and society (Chardnarumarn et al., 2021).

This was fortunate for Thailand as the subsequent COVID 19 pandemic has been catastrophic across all sectors of the economy, with all forms of education at all levels having to find a way to make online learning a reality sooner than later. Proof of this can be found in the MOE's newest policies in which online education is a critical element in providing student safety under the' New Normal.' Moreover, the essential nature of *Distance Learning Television* has been reemphasized, which is a form of asynchronous learning.

The MOE has also stated the need for teachers to shift their teaching programs to two-way or synchronous communications for secondary school level students (grades 7–12) while simultaneously providing support to students by various online platforms. Furthermore, teachers must enhance their ICT skills and develop online assessment processes.

Also, the Thai MOE has indicated a radical shift in how education budgets will be used in the future. These shifts will entail funds being moved from traditional schools and allocated to online course development, online teaching, and ICT digital devices to improve online learning effectiveness (Chardnarumarn et al., 2021).

Given these significant changes to Thailand's present and future student education development under what is now known as the 'New Normal,' we felt an imperative need to undertake a study to investigate how students perceived these changes and how satisfied they were. Therefore, this study undertook an extensive literature review presented in Section 2. We identified seven possible constructs that could affect online education student satisfaction. Moreover, ten hypotheses were conceptualized, which led to a 53 item questionnaire. After obtaining ethics clearance from our university, experts reviewed the survey instrument, followed by a pilot test on 30 students not involved with the subsequent study. Using multi-stage and simple random, 270 students were eventually selected and invited to answer the questionnaire across nine Thai provinces.

After the collection of the questionnaires and their audit, a confirmatory factor analysis (CFA) and goodness-of-fit (GOF) assessment were performed (Section 4). After that, structural equation modeling (SEM) was undertaken. Section 5 contains a detailed discussion, followed by the conclusion (Section 6), the potential study limitations and future suggestions, and finally, contribution to the research.

## 2 Literature review

### 2.1 Performance expectancy (PE)

In their analysis of technology use models, Venkatesh et al. (2003) identified *performance expectancy* as how an individual feels about their use of technology and to what degree the technology use will improve their job experience. Also, according to previous research on online learning (Jongkolthanalarp et al., 2021; Lakhal et al., 2013; Mendoza et al., 2017), students expect that online learning use will help them understand the lesson better, and they believe that online education will provide them with more accurate knowledge. Also, students believe that adopting online learning will improve their academic performance. Consequently, studies suggest that they think their level of satisfaction will increase if what they learn is what they expected.

Moreover, degrees of performance expectation can be influenced by several variables (Davis et al., 1992; Venkatesh et al., 2003). These can include *intrinsic motivation*, *extrinsic motivation*, and *perceived ease of use* (Fagan et al., 2008). It should also be noted here that *intrinsic motivation* is sometimes discussed in terms of *internal motivation*, and *extrinsic motivation* is sometimes referred to as *external motivation*. However, in developmental psychology, intrinsic motivation is an essential concept central to an individual's spontaneous curiosity and exploration (Oudeyer and Kaplan, 2009). Also, intrinsic motivation is the psychological force that motivates an individual to do something for the pleasure of their feelings rather than for the sake of achieving some other goal (Eom and Ashill, 2016), with most students reported to benefit from intrinsic motivators. Involvement, interest, challenge, and social interaction are some of these elements.

On the other hand, extrinsic motivation motivates a person to accomplish something toward a goal to obtain a different consequence, such as a prize or praise (Eom and Ashill, 2016). Scholars have reported that both intrinsic and extrinsic motivation impact students' learning outcomes (Fagan et al., 2008; Williams and Williams, 2011). Therefore, three observed variables were selected as influencing factors on a student's *performance expectancy* (PE). These included *intrinsic motivation* (x1), *extrinsic motivation* (x2), and *perceived benefit* (x3). Finally, the following two hypotheses are presented for the study:

H1 *Performance Expectancy (PE) directly affects Behavioral Intention* (BI).

H2 *Performance Expectancy* (PE) directly affects *Student Satisfaction* (SS).

### 2.2 Effort expectancy (EE)

Another important aspect of technology use is EE, or the perceived ease of use (Venkatesh et al., 2003), which can be thought of as how technology use impacts a consumer's *behavioral intention* (BI). The perceived ease of use or 'usability' concept was demonstrated in Greece from research from Papakostas et al. (2021), who investigated how artificial reality (AR) simulation could be applied in simulation in vocational education and training (VET). In their groundbreaking research, the authors examined how artificial reality and vocational education and training could be merged for industrial manufacturing training. The findings showed that perceived enjoyment and system quality were strong predictors of the proposed model and evaluated pedagogical affordance and technological innovation simultaneously.

Therefore, students potentially will have greater satisfaction with online learning if the system is straightforward to use, and they have to put less effort into comprehending and utilizing it (Lakhal et al., 2013; Jongkolthanalarp et al., 2021). Moreover, Im et al. (2011) have reported that the impact of EE is more substantial on BI in nations such as the US over other countries such as Asian nations such as South Korea.

Course design has also significantly influenced learning, both in traditional and online settings (Lee, 2014). Sun and Chen (2016) argued that a flawless course design that encouraged teacher-student interaction led to effective utilization of online education and well-prepared teachers. Furthermore, Liu et al. (2010) discovered that online course creation is an essential predictor of SS.

Therefore, two observed variables were selected as influencing factors on a student's *effort expectancy* (EE). These included *perceived benefits* (x4) and *course design* (x5). Finally, the following hypothesis is presented for the study:

H3 Effort Expectancy (EE) directly affects Actual Use (AU).

### 2.3 Social influence (SI)

The degree to which a person considers a peer's perception of a system's use as necessary is referred to as *social influence* (Venkatesh et al., 2003), with various scholars pointing out the importance of SI on student retention in post-secondary education (Fleming et al., 2017). Im et al. (2011) have also reported that when it comes to technology adoption, users in more collectivistic and higher power distance cultures will be affected by others.

The term ‘*subjective norm*’ refers to a mix of close individuals' perceived expectations that are important to the individual and thus ambitious to meet their expectations (Davis, 1989; Lakhal et al., 2013). Individuals' absorption of the subjective culture of reference groups and particular interpersonal agreements established with others in specific social settings are characterized as ‘*social factors’* (Thompson et al., 1991; Lakhal et al., 2013). Therefore, two observed variables were selected as influencing factors on a student's *social influence* (SI). These included *subjective norms* (x5) and *social factors* (x6). Finally, the following hypothesis is presented for the study:

H4 *Social Influence* (SI) directly affects *Behavioral Intention* (BI).

### 2.4 Learner interaction (LI)

Due to the physical distances between students and teachers, interactivity has long been seen as one of the most essential elements of online education (Kuo et al., 2013). According to multiple scholars, little interaction between students and teachers is a significant source of concern (Kuo et al., 2013; Hebebci et al., 2020; Baber, 2020).

An investigation into these issues by Moore (1989) proposed a three-tiered interaction paradigm in which *learner-learner interaction* (LLI), *learner-instructor interaction* (LII), and *learner-content interaction* (LCI) were classified. The author then defined *learner-learner interaction* as the communication between students who may share course-related knowledge, information, or opinions, and *learner-instructor interaction* as the interactions between instructors and students. At the same time, *learner-content interaction* was the process of students elaborating, learning, and commenting on course information. Later studies discovered that these three interactions were substantial predictors of SS in online learning (Alqurashi, 2018; Bisht et al., 2020; Kuo et al., 2013; Parahoo et al., 2015).

Furthermore, Bisht et al. (2020) revealed that a lack of *learner-learner interaction* with classmates and LLI with faculty members was identified as a barrier to online education, suggesting that it may predict SS. Alqurashi (2018) also discovered that *learner-content interaction* is the most crucial factor influencing SS among the three forms of interaction. When the learning environment goes online, children generally spend a significant amount of time processing information, digesting knowledge, and learning on a digital screen. This form of self-learning from the material may necessitate engagement to learn and be satisfied with the content.

Also, although *learner-learner interaction* continues to be a significant predictor of SS (Baber, 2020), students are still likely to be satisfied if they have high-quality interactions with their instructors (Alqurashi, 2018). Teachers' prompt responses and comments are essential because there is no face-to-face connection in online education. Therefore, three observed variables were selected as influencing factors on a student's *learner interaction (LI).* These included *learner and learner interaction* (x8), *learners and teachers' interaction* (x9), *learners and learning content interaction* (x10). Finally, the following two hypotheses are presented for the study:

H5 *Learner Interaction* (LI) directly affects *Behavioral Intention* (BI).

H6 *Learner Interaction* (LI) directly affects *Student Satisfaction* (SS).

### 2.5 Facilitating conditions (FC)

We believe that ICT is one of the most essential elements in conducting online education courses is well supported in the global literature. Without digital devices and Internet connectivity, learning portals such as Khan Academy could not exist. Beyond the ‘wow’ factor in the production of the online courses, students are allowed to work at a pace that is slow or fast as they need (Khan, 2016). Also, the online courses are focused on ‘mastery’ of the concepts and materials, not test scores.

Venkatesh et al. (2003) implied that *facilitating conditions* are how well students believe their school's ICT infrastructure can meet their online needs. Furthermore, online learning involves a range of online activities in addition to the actual online classroom (Kuo et al., 2013; Rasmitadila et al., 2020). There also needs to be a well-established internet infrastructure (Zhou et al., 2020), which is necessary to provide a comprehensive and successful online learning experience. Moreover, inconsistent and unreliable Internet access significantly influences students' satisfaction (and teachers) in their use of online education, as is the necessary Internet bandwidth for online students to complete required course assignments (Kuo et al., 2013).

Mendoza et al. (2017) also reported that Internet connectivity limitations could be a barrier to successful online education in terms of both use and satisfaction. Therefore, students and teachers should ensure reliable and sufficient Internet connectivity and an appropriate physical learning environment before the commencement of an online learning session (Nonthamand et al., 2021).

Furthermore, Hebebci et al. (2020) has highlighted that a lack of technological preparedness is one of the most significant downsides of online education activities. In India, Nambiar (2020) observed that technical issues (such as poor Internet signals, poor video quality, and login difficulties of various courses) were the most problematic parts of their online classes for over 50% of the students in the study's sample. Also, students expect instructors or other staff members to assist them with technological issues. Students, like instructors, needed to be taught how to utilize an online program or media to participate in an online class (Nambiar, 2020).

Moreover, Dhawan (2020) and Faize and Nawaz (2020) identified technological difficulties as significant challenges students face in online education. Several other researchers have also suggested that improved technology is a critical component in making online education effective (Faize and Nawaz, 2020; Hebebci et al., 2020; Nonthamand et al., 2021; Sun and Chen, 2016). Therefore, two observed variables were selected as influencing factors on a student's *facilitating conditions (FC).* These included *ICT infrastructure* (x11) and *Internet connectivity* (x12). Finally, the following two hypotheses are presented for the study:

H7 *Facilitating Conditions* (FC) directly affects *Actual Use* (AU).

H8 *Facilitating Conditions* (FC) directly affects *Student Satisfaction* (SS).

### 2.6 Behavioral intention (BI) and actual use (AU)

It is said that behavioral intention captures the motivating elements that influence behavior. The intention measures how far individuals are willing to act and how much work they are prepared to put in (Lakhal et al., 2013). Also, according to Venkatesh et al. (2003), behavioral intention positively impacted actual usage.

Actual usage can relate to the frequency, type, and duration with which an individual makes use of the capabilities of ICT and a digital device (Aldholay et al., 2018 DeLone and McLean, 2016; Kim et al., 2007), with *actual use* indicating the frequency and length of use in online learning (Kim et al., 2007). Additionally, various studies have reported that *actual use* has a significant effect on *student satisfaction* when they the Internet and ICT (Aldholay et al., 2018; Hou, 2012; Isaac et al., 2017). Therefore, two observed variables were selected as influencing factors on a student's *learner interaction (LI).* These included *frequency use prediction* (y1) and *plan to use* (y2). For *actual use* (AU), we also used two observed variables, which included frequency of use (y3) and usage time (y4). Finally, the following two hypotheses are presented for the study:

H9 *Behavioral Intention* (BI) directly affects *Actual Use* (AU).

H10 *Actual Use* (AU) directly affects *Student Satisfaction* (SS).

### 2.7 Student satisfaction (SS)

*Student satisfaction* is a crucial measure of how well students are doing in their classes, leading to different outcomes, such as student retention and course quality (Alqurashi, 2018; Bolliger and Martindale, 2004; Fleming et al., 2017; Kuo et al., 2013). According to studies, satisfied customers are loyal, and satisfied students will most likely attend another session by the same teacher (Devinder and Datta, 2003). Therefore, educational institutions must view *student satisfaction* as a valuable asset as students are more likely to talk about their experiences positively and return as alumni (Parahoo et al., 2015).

Furthermore, a variety of factors influence *student satisfaction* in traditional classrooms, including student characteristics, educational quality, and utility, curriculum and instruction, student life, interaction in both face-to-face and online classes, technological features, their learning styles, support services, and, on rare occasions, demographic characteristics (Yilmaz, 2017). On the other hand, online education provides a unique set of problems because students may never visit a physical location and struggle to form relationships with their peers (Bolliger and Martindale, 2004; Parahoo et al., 2015; Yukselturk and Yildirim, 2008). Finally, multiple studies have shown that a variety of factors impact student satisfaction, including *student retention* (y5) and *course quality* (y6). Therefore, from the brief literature overview, we present Table 1.

**Table 1:** The questionnaire constructs, their observed variables, item totals, and supporting theory.

Constructs	Observed variables	Items 53	Supporting theory
Performance Expectancy (PE)	intrinsic motivation (x1) extrinsic motivation (x2) perceived benefit (x3)	3 3 3	(Davis et al., 1992; Eom and Ashill., 2016; Jongkolthanalarp et al., 2021; Krouska et al., 2021; Lakhal et al., 2013; Mendoza et al., 2017; Teo et al., 2019; Venkatesh el al., 2003; Williams and Williams, 2011).
Effort Expectancy (EE)	perceived ease of use (x4) course design (x5)	3 4	(Kanetaki et al., 2021; Lakhal et al., 2013; Lee, 2014; Liu et al., 2010; Jongkolthanalarp et al., 2021; Papakostas et al., 2021; Sun & Chen., 2016; Venkatesh et al., 2003; Woodworth et al., 2015).
Social Influence (SI)	subjective norms (x6) social factors (x7)	3 3	(Baki et al., 2021; Davis, 1989; Im et al., 2011; Lakhal et al., 2013; Thompson et al., 1991; Venkatesh, 2000; Venkatesh et al., 2003).
Learner Interaction (LI)	learner and learner interaction (x8) learners and teachers' interaction (x9) learners and learning content interaction (x10)	3 3 3	(Alqurashi, 2018; Asmuni et al., 2012; Baber, 2020; Bisht et al., 2020; Hebebci et al., 2020; Kanetaki et al., 2021; Krouska et al., 2021; Kuo et al., 2013; Moore, 1989; Parahoo et al., 2015; Pimdee and Leekitchwatana, 2022; Teo et al., 2019; Yawson and Yamoah, 2020).
Facilitating Conditions (FC)	ICT infrastructure (x11) Internet connectivity (x12)	3 3	(Bisht et al., 2020; Chardnarumarn et al., 2021; Darawong and Widayati, 2021; Dhawan, 2020; Faize and Nawaz, 2020; Hebebci et al., 2020; Kuo et al., 2013; Mendoza et al., 2017; Nambiar, 2020; Nonthamand et al., 2021; Oranop, 2016; Rasmitadila et al., 2020; Ruenphongphun et al., 2021; Venkatesh et al., 2003; Zhou et al., 2020).
Behavioral Intention (BI)	frequency use prediction (y1) plan to use (y2)	2 3	(Baki et al., 2021; Lakhal et al., 2013; Teo et al., 2019; Venkatesh et al., 2003).
Actual Use (AU)	frequency of use (y3) usage time (y4)	2 4	(Aldholay et al., 2018; DeLone and McLean, 2016; Hou, 2012; Isaac et al., 2017; Kim et al., 2007; Papakostas et al., 2021; Thongsri et al., 2019).
Student Satisfaction (SS)	student retention (y5) course quality (y6)	2 3	(Alqurashi, 2018; Bolliger and Martindale, 2004; Devinder and Datta, 2003; Fleming et al., 2017; Harsası and Sutawıjaya, 2018; Kuo et al., 2013; Parahoo et al., 2015; Yilmaz, 2017; Yukselturk and Yildirim, 2008).

### 2.8 Objectives of the research

1. To explore the construct and observed variable interrelationships influencing online learning *student satisfaction* using a *structural equation model.*
2. To assess the proposed model's fit by using a *goodness-of-fit* and *confirmatory factor analysis* before the SEM.
3. To make recommendations to schools and administrators on which components lead to *student satisfaction* and online learning system efficiency.

## 3 Materials and methods

### 3.1 Ethics clearance

The primary researcher attended a 6-h course titled *Good Clinical Practice (2021)* before the commencement of the study. During this session, 12 modules were covered concerning conducting an ethical research study. Using this valuable information, we then presented our research plan methodology to our university' *Human Ethics Committee*, from which we obtained approval and a suggestion to receive an informed consent form from each expert, provincial teacher assistant, pilot-test and student participant, assuring each individual's anonymity (Chuenban et al., 2021).

### 3.2 Population and sample size

This study used a quantitative research technique to reach the research objectives, which were the investigation of factors that influence *student satisfaction* with online learning. The survey commenced in June 2021 and was completed in August 2021. The population included 371,845 students from Thailand's extra-large public high schools (OBEC, 2021). The sample size determination evaluated suggested multiple sample size collection methodologies depending on complexity and observed variables. Osborne and Costello (2004) have also further added that in studies using ***confirmatory factor analysis***, a ratio of 10–20 questionnaires should be collected for each observable variable. However, although there are numerous studies supporting a 10:1 ratio for a CFA (Gorsuch, 1997; Kline, 1979; Markus, 2012), the authors increased the sample size objective to 360 (Table 2). This is consistent with other scholars who have stated that CFA/SEM research should have sample sizes of 200 or more depending on the complexity of the model (Cattell, 1978; Everitt, 1975; Guilford, 1954; Hair et al., 2016; Schumacker and Lomax, 2016). Therefore, to assure reliability we increased the sample size target threshold to 360 questionnaires, which met the often cited sample size ratio of 10:1 and a minimum sample size of 200 total questionnaires. This was done due to the know issues of incomplete surveys due to non-response error (Dillman et al., 2013; Millar and Dillman, 2012) and low response rates (Pielsticker and Hiebl, 2020). After all the questionnaires were collected and reviewed, 270 were judged to be complete enough for use in the study's analysis (Table 2) (Alguacil et al., 2021; Cattell, 1978).

**Table 2:** Online study student satisfaction sampling processes (*n* = 270).

Region	Schools (Province)	Pop./Quest.	Samples (Gender)				Total
			male		female		
			Count	%	Count	%	Count
Northern	Nareerat (Phrae)	1,398/35	7	2.59	23	8.52	30
Central	Ayutthaya Wittayalai (Ayutthaya)	2,197/40	16	5.93	14	5.19	30
	Prommanusorn (Phetchaburi)	1,575/35	15	5.56	15	5.56	30
	Sakaew (Sakaew)	1,358/35	11	4.07	19	7.04	30
	Thammasat Khlongluang Wittayakom (Pathum Thani)	2,428/45	11	4.07	19	7.04	30
Northeast	Narinukun (Ubon Ratchathani)	1,905/40	7	2.59	23	8.52	30
	Lam Plai Mat (Buriram)	1,398/40	16	5.93	14	5.19	30
	Kalasin Phitthayasan (Kalasin)	1,758/45	8	2.96	22	8.15	30
South	Wichienmatu (Trang)	1,496/45	11	4.07	19	7.04	30
Totals/%		15,513/360	102	37.78	168	62.22	270

Note*.* Pop. Is the student population for each province in the targeted class levels. Quest. is the number of targeted questionnaires for each province.

Also, to assure statistical validity after evaluation of the various criteria, we decided to sample students from four separate Thai regions from which we used multi-stage random sampling to select nine extra-large secondary schools in nine provinces (Leekitchwatana and Pimdee, 2021; Prasittichok and Klaykaew, 2022). After that, we utilized simple random sampling to choose students in each region/school from an extra-large public high school.

### 3.3 Data collection

Due to the complexities of doing face-to-face surveys during the Covid-10 pandemic lockdowns, we relied on a network of teachers in nine Thai provinces, including *Phrae, Ayutthaya*, *Phetchaburi, Sakaew, Pathum Thani, Ubon Ratchathani, Buriram, Kalasin, and Trang* (Table 2). Using these teachers as a base for the provincial studies and student support, multi-stage random sampling was used to identify and invite the study's participants, who were then asked their opinions concerning their satisfaction with their experiences using their online learning system during the COVID-19 pandemic (Table 2). The Google Form online tool was used to obtain each student's Thai language response to the questionnaire (Ruenphongphun et al., 2021).

### 3.4 The Questionnaire's design

The student questionnaire contained two sections in which Section 1 asked 14 items about each student's personal, school, and online experiences, followed by one open-ended item (Table 3). In Section 2, 53 items covered the study's eight latent variables and 18 observed variables (Table 1). The questionnaire also used a five-level scale with ‘5’ as an opinion indicator that the student strongly agreed (4.50–5.00), while ‘1’ indicated that the student strongly disagreed (1.00–1.49) (Ruenphongphun et al., 2021).

**Table 3:** Summary of student information (*n* = 270).

Type of information	A summary of results
Gender	Females made up the majority of the students (62.20%).
Student age	There was a near-even match in the survey between 17-year-old students (34.80%) and 17-year-old students (32.20%), while 18.10% said they were 15.
Grade of the student (High School/Secondary School)	The majority of the high school students stated they were in Grade 11 (35.20%), while 33.30% said they were in Grade 12, and the remaining 31.50%.reporting they were in Grade 10.
Place (province) of living	Each student attended an extra-large high school in Thailand, which was equally divided into 30 students from each province for sampling purposes.
How many online learning systems do you use at school?	87.80% of the students reported using more than one online learning system, while the remaining 12.20% mentioned that they used only one online learning system in their school.
Is your school's online learning system able to study asynchronously (e.g., Line and WhatsApp messaging and e-mail)?	More than half (50.40%) of the students from this survey answered that their schools offered them the ability to study asynchronously, while 49.60 % of the students answered that their schools did not provide asynchronous study.
In addition to studying online with your school, do you have extra online tuition with other places?	38.50% of the students reported they had additional online tuition costs with other places, while 61.50% said they studied online only with their school.
How many hours do you study online each day?	45.60% of the students reported they studied online 7–8 h per day, while 33.30% said they learned online 4–6 h per day, with the remaining 13.30% reporting they studied online 8 h or more.
What is your family's average monthly income?	10,001 baht to 25,000 baht per month ($300-$750) was the answer selected most (34.80%). This was followed by less than 10,000 baht (27.00%) and 25,001 baht to 40,000 baht (20.40%).
Whom do you live with?	68.50% of the students reported they lived with both of their parents, followed by 16.30% who declared they lived with either their mother or their father, while 14.40% of the students answered that they lived with a relative or others.
What devices do you primarily use for online learning?	One hundred sixty-five students (61.10%) used smartphones as their primary digital, whereas 17.80% connected online with a laptop. The third most used device was a computer desktop (13.00%).
Does the digital device in the previous question already exists or needs to be purchased new?	Two hundred forty-two students, or 89.60%, already had their digital device to connect online, whereas 10.40% had to find, buy, or borrow a new one.
What do you use to connect to the Internet? Your phone SIM or a Wi-Fi signal?	One hundred eighty-six students (68.90%) reported using a Wi-Fi signal for their online learning, while 31.10% said they used their phone SIM to go online.
Overall, how much does online learning cost?	199 students (73.70%) stated that online learning cost them 0–5,000 baht, while 50 students (18.50%) reported their cost was 5,001–10,000 baht. A much smaller group of 3.30% answered 10,001–15,000 baht.
How many additional monthly expenses do you have for online learning?	Ninety-nine students (36.70%) reported paying an additional monthly expense of 101–500 baht, while 76 students (28.10%) reported paying an additional monthly fee of 501–1,000 baht. Finally, 58 students (21.50%) said they spent an additional monthly expense of more than 1,000 baht.
What would you do to improve online learning in Thailand? (Open-ended)	Sixty students (22.22% of the total respondents) provided responses, from which some were concerned with online course design problems, while others gave a political-related answer.

Note: On 27 January 2022, the baht to the dollar was 30.12 baht to $1.00USD.

### 3.5 Research instrument quality assessment

After the questionnaire's design, a *content validity* (CV) assessment was undertaken for the questionnaire (Chuenban et al., 2021). It has been suggested that the strength and *validity* of the research design come from how accurately variables are selected and measured (Wannapiroon and Pimdee, 2022).

For this process, many researchers will use a panel of experts drawn from related fields to the study. In our case, we contacted five experts who volunteered to participate in the content analysis. All five experts had obtained their Ph.D. and had a minimum of 10 years experience with their respective fields of expertise and teaching.

From that, we used the Cronbach's Alpha α measurement values suggested by George and Mallery (2010) and determined that the average coefficient = 0.83, which is classified as ‘good’ (Table 5). The data analysis was calculated using descriptive statistics, entailing the mean average ($\bar{x}$), frequency, and percentage, and a SEM to assess the ten hypotheses.

### 3.6 The questionnaire pre-test and measurement of validity

A *pilot test* was undertaken before the actual surveys in which 30 students participated. These students were not part of the final survey, which helped the researchers determine each item's questionnaire relevance and clarity (Converse and Presser, 1986). .

## 4 Results

**Figure 1:**
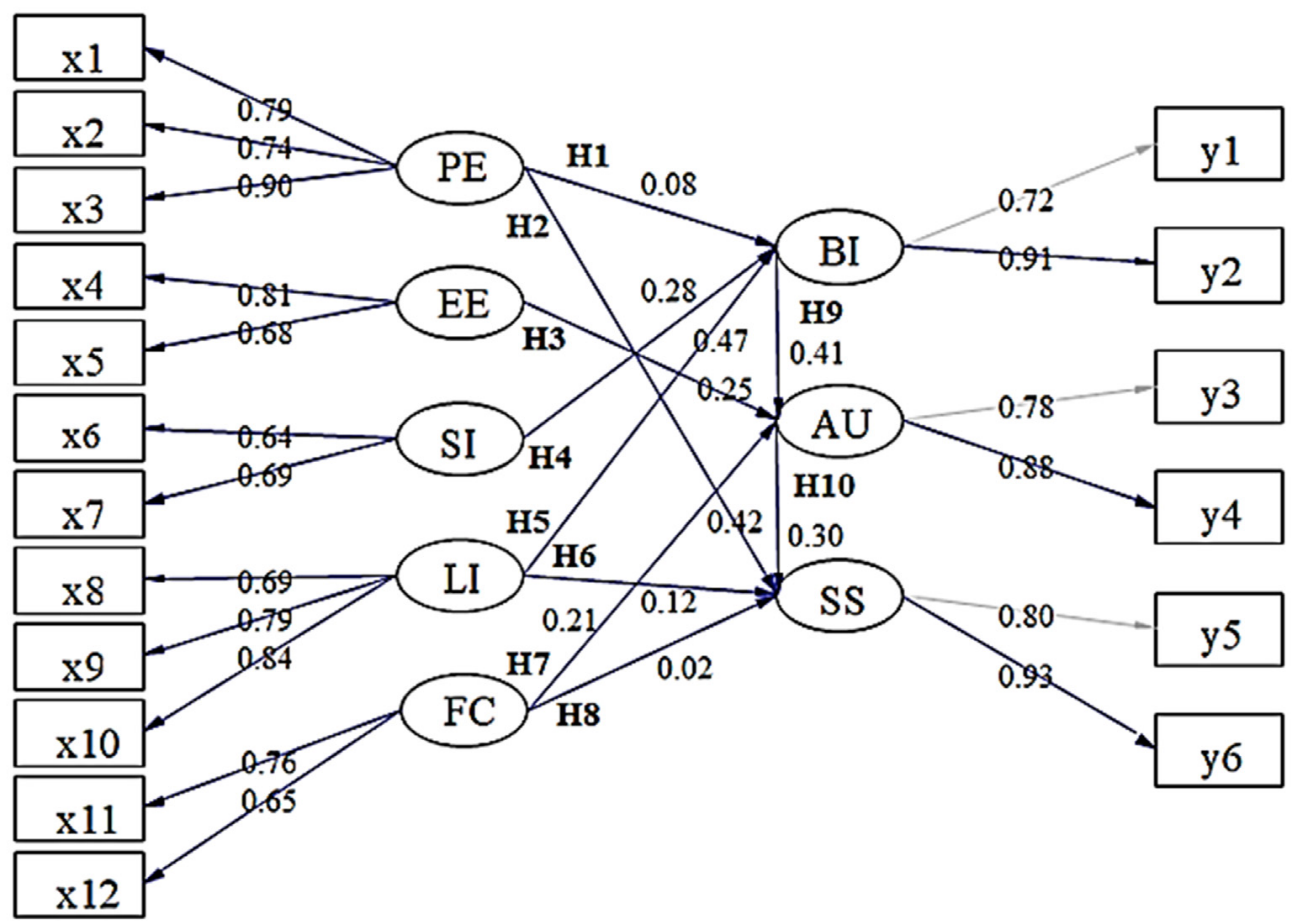
Final results from the LISREL 9.1 SEM. Chi-Square = 102.02, df = 88, *p*-value = 0.14569, RMSEA = 0.024. Please see Table 5 for the latent variable and observed variable descriptions (y1, x4, etc.).

During the COVID-19 pandemic in Thailand, researchers surveyed 270 high-school students across nine provinces, revealing multiple aspects concerning their satisfaction in using an online learning system. The research findings present the personal information from the 270 students in Table 3, while Figure 1 and Table 8 display the results from the SEM of the variables affecting online study student satisfaction.

### 4.1 The research results for student information (n = 270)

Table 3 shows that females comprised most of the high school student respondents, accounting for 62.20% of the total, with 34.80% 16 years old. The respondents were divided into 30 students from nine Thai provinces for sampling purposes, including *Kalasin, Trang, Buriram, Pathum Thani, Phetchaburi, Phrae, Sa Kaeo Ayutthaya*, and *Ubon Ratchathani*.

Furthermore, 87.80% of students reported using more than one online learning system at their school, while 50.40% of the respondents said their institutions allowed them to study asynchronously (e.g., Line and WhatsApp messaging and e-mail). However, 38.50% reported they paid additional online tuition to learn online with their institution. When asked how many hours they spent studying online, 68.9% of the students said they spent at least 7 h per day.

In addition to personal information, 34.8% reported that their family's monthly income was 10,001 baht to 25,000 baht ($300-$750). Furthermore, 61.1% of the students said they used smartphones to study online, while 89.60% reported that they already had a digital device when their online classes commenced. Concerning Internet coverage, 68.9% said they connected to the Internet using a Wi-Fi signal, whereas the remainder used their telephone's SIM. Because most students already had their equipment for studying, the online learning cost for most students was from 0 - 5,000 baht.

### 4.2 Goodness-of-fit testing (GOF) results

Before the SEM, a *goodness-of-fit* was undertaken (Table 4) with various statistical software packages using different *goodness-of-fit* nomenclature and criteria. However, LISREL 9.1 commonly uses χ2/df. As with most indices, there are multiple criteria used. However, using similar studies from other authors, we embraced standards in which χ2/df ≤ 2.00 (Sahoo, 2019). Also, frequently cited scholars are Jöreskog et al. (2016), who recommends that in LISREL modeling that the goodness of fit index (GFI) ≥ 0.90, comparative fit index (CFI) ≥ 0.95, *p* ≥ 0.05, and root mean square error of approximation (RMSEA) ≤ 0.05 while Schumacker and Lomax (2016) suggest that values for the normed fit index (NFI) ≥ 0.90, AGFI ≥0.90, root mean square residual (RMR) ≤ 0.05, and standardized root mean square residual (SRMR) ≤ 0.05. Therefore, as shown in Table 4, all GOF values significantly exceeded the suggested minimal GOF criterion, implying that the model fit was excellent (Cangur and Ercan, 2015).

**Table 4:** The GOF of factors affecting online study student satisfaction (SS).

	χ^2^	Df	χ^2^/Df	GFI	AGFA	CFI	NFI	RMSEA	RMR	SRMR
Criteria	n/a	n/a	≤2.00	≥0.90	≥0.90	≥0.95	≥0.90	≤0.05	≤0.05	≤0.05
Values	102.02	88	1.16	0.96	0.92	0.99	0.99	0.02	0.03	0.03
Results	n/a	n/a	Valid	Valid	Valid	Valid	Valid	Valid	Valid	Valid

Note: χ^2^ = Chi-square, Df = Degrees of freedom.

### 4.3 CFA results

Table 5 presents the data collected from the CFA. Once again, we note the 'good' values from 0.79 - 0.89 with an average value of 0.83 (George and Mallery, 2010). Moreover, the loading factors for each variable are substantial as they all exceed that suggested value of are also ≥0.5 (Hooper, 2008). Likewise, Hooper et al. (2008) also recommend that R^2^ values be less than ≤0.20. Also, Ab Hamid et al. (2017) have suggested that composite reliability (CR)/Cronbach α values from 0.60 to 0.70 are acceptable in exploratory research. With the range of values for R^2^ falling between 0.41 - 0.79 and CR values 0.60–0.86, further model strength is established. Finally, another standard recommendation for the model's fit validity is the use of the average variance extracted (AVE), which for this study were 0.43–0.76. It should be noted that Fornell and Larcker (1981) have stated that if AVE ≤0.5 but CR values are ≥0.6, convergent validity (CV) is acceptable. The construct CV is still acceptable if AVE is less than 0.5, but CR is ≥0.6.

**Table 5:** Overview of the study's data analysis for the constructs and the observed variables.

Constructs	α	Observed variables	Loading	R^2^	CR	AVE
Performance Expectancy (PE)	0.85	intrinsic motivation (x1)	0.80	0.63	0.84	0.63
		extrinsic motivation (x2)	0.69	0.47		
		perceived benefit (x3)	0.89	0.79		
Effort Expectancy (EE)	0.84	perceived ease of use (x4)	0.82	0.67	0.71	0.55
		course design (x5)	0.66	0.43		
Social Influence (SI)	0.79	subjective norms (x6)	0.64	0.41	0.60	0.43
		social factors (x7)	0.67	0.45		
Learner Interaction (LI)	0.89	learner and learner interaction (x8)	0.68	0.47	0.82	0.60
		learners and teachers' interaction (x9)	0.78	0.60		
		learners and learning content interaction (x10)	0.86	0.73		
Facilitating Conditions (FC)	0.79	ICT infrastructure (x11)	0.79	0.62	0.69	0.53
		Internet availability (x12)	0.66	0.44		
Behavioral Intention (BI)	0.84	frequency use prediction (y1)	0.79	0.63	0.81	0.68
		plan to use (y2)	0.86	0.74		
Actual Use (AU)	0.82	frequency of use (y3)	0.81	0.66	0.82	0.69
		usage time (y4)	0.85	0.72		
Student Satisfaction (SS)	0.85	student retention (y5)	0.78	0.61	0.86	0.76
		course quality (y6)	0.95	0.91		

### 4.4 Mediation effects on the exogenous latent variables and endogenous latent variables

The analysis showed that all the model's causal variables positively affected SS, which, when combined, had an R^2^ of 54% (Table 6). Moreover, the TE values for the latent variables from when ranked were *performance exp*ectancy (0.43), *actual use* (0.30), *learner interaction* (0.18), and *behavioral intention* (0.12), *effort expectancy* (0.08), *facilitating conditions* (0.08), and finally, *social influence* (0.04). Moreover, there was a moderately strong effect between BI and LI (0.47) and *student satisfaction* and *performance exp*ectancy (0.42).

**Table 6:** Summary of DE, IE, and TE of each construct.

Endogenous Latent Variables	Effects	R^2^	Exogenous Latent Variables						
			PE	EE	SI	LI	FC	BI	AU
Behavioral Intention (BI)	DE	0.61	0.08	-	0.28∗	0.47∗∗	-		
	IE		-	-	-	-	-		
	TE		0.08	-	0.28∗	0.47∗∗	-		
Student Satisfaction (SS)	DE	0.54	0.42∗∗	-	-	0.12	0.02	-	0.30∗∗
	IE		0.01	0.08	0.04	0.06	0.06	0.12∗∗	-
	TE		0.43∗∗	0.08	0.04	0.18	0.08	0.12∗∗	0.30∗∗
Actual Usage (AU)	DE	0.53	-	0.25∗	-	-	0.21∗	0.41∗∗	
	IE		0.03	-	0.12	0.19∗	-	-	
	TE		0.02	0.25∗	0.12	0.19∗	0.21∗	0.41∗∗	

∗Sig. < .05, ∗∗Sig. < .01.

### 4.5 Testing results of the construct correlation coefficients

In Table 7, we found strong correlations based on interpretations commonly used, which suggests that when Pearson's *r* values are 0.50–1, the correlation is strong (Chuenban et al., 2021). Also, further confirmation of validity is obtained when standardized factor loading values ≥0.60 (0.64–0.95).

**Table 7:** Testing results for construct correlation coefficients.

Constructs	BI	AU	SS	PE	EE	SI	LI	FC
BI	**1**							
AU	0.712∗∗	**1**						
SS	0.598∗∗	0.649∗∗	**1**					
PE	0.671∗∗	0.610∗∗	0.713∗∗	**1**				
EE	0.735∗∗	0.686∗∗	0.695∗∗	0.882∗∗	**1**			
SI	0.720∗∗	0.679∗∗	0.621∗∗	0.724∗∗	0.901∗∗	**1**		
LI	0.762∗∗	0.672∗∗	0.686∗∗	0.835∗∗	0.885∗∗	0.819∗∗	**1**	
FC	0.556∗∗	0.596∗∗	0.501∗∗	0.534∗∗	0.628∗∗	0.745∗∗	0.652∗∗	**1**
CR	0.803	0.817	0.858	0.853	0.716	0.614	0.818	0.665
AVE	0.673	0.691	0.752	0.661	0.559	0.443	0.602	0.500
$\sqrt{\text{A}\text{V}\text{E}}$	0.820	0.831	0.867	0.813	0.748	0.665	0.775	0.707

∗∗*p* ≤ .01.

### 4.6 Final hypotheses testing results

Table 8 and Figure 1 detail the ten hypotheses testing results, from which seven were supported (S), and three were not supported (NS). Moreover, significant strength was found in the H5 relationship between LI and BI (*r =* 0.47, t-value = 3.20∗∗), followed by H2 and the relationship from *performance exp*ectancy to *student satisfaction* (*r =* 0.42, t-value = 2.63∗∗), and H9 the relationship from BI to AU (*r =* 0.41, t-value = 3.87∗∗). Hair et al. (2016) has also suggested that CVs are acceptable when t-values ≥ 1.96.

**Table 8:** Results of the hypotheses testing.

Hypotheses	*r*	t-value	Validity
H1: Performance Expectancy (PE) directly affects Behavioral Intention (BI).	0.08	0.65	NS
H2: Performance Expectancy (PE) directly affects Student Satisfaction (SS).	0.42	3.20∗∗	S
H3: Effort Expectancy (EE) directly affects Actual Use (AU).	0.25	2.20∗	S
H4: Social Influence (SI) directly affects Behavioral Intention (BI).	0.28	2.03∗	S
H5: Learner Interaction (LI) directly affects Behavioral Intention (BI).	0.47	2.63∗∗	S
H6: Learner Interaction (LI) directly affects Student Satisfaction (SS).	0.12	0.81	NS
H7: Facilitating Conditions (FC) directly affects Behavioral Intention (BI).	0.21	2.40∗	S
H8: Facilitating Conditions (FC) directly affects Student Satisfaction (SS).	0.02	0.21	NS
H9: Behavioral Intention (BI) directly affects Actual Use (AU).	0.41	3.87∗∗	S
H10: Actual Use (AU) directly affects Student Satisfaction (SS).	0.30	3.56∗∗	S

∗*p* ≤ 0.05, ∗∗*p* ≤ 0.01, S = supported, NS = not supported.

## 5 Discussion

This section presents a discussion of the factors determined to affect Thai online study student satisfaction during the ongoing COVID-19 pandemic in Thailand. Moreover, it was determined that the R^2^ 54% and the total effect of *performance expectancy's* perceived benefit (x3), intrinsic motivation (x1), and extrinsic motivation (x2) were significant, with loading factors of 0.89, 0.80, and 0.69, respectively. Finally, the hypotheses testing results from the SEM revealed three moderately strong correlations and four weak correlations (Table 8), with three of the study's ten hypotheses determined to be not supported.

### 5.1 Student information

In Table 1, we note the higher percentage of female to male students within the study. This is consistent with many other Thai studies for this age group in secondary school, which has been speculated to be due to male students transferring over to vocation education and the need for males to work on their parents' farms as they become older. Therefore, the repercussions of this gender imbalance have the potential to affect online learning outcomes as (Venkatesh et al., 2003) confirmed the importance of gender in technology acceptance with females focused on the *perceived ease of use* (x4) over a male's focus on *perceived benefit* (x3). In our study these items were found to be very important as x4 = 0.82 and x3 = 0.89.

Also, from Table 1, we note what appears to be an extraordinarily high number of hours of online time being reported by the students, with 45.60% reporting they studied 7–8 h per day, with 13.30% reporting they studied online 8 h or more. This number of hours is consistent with other online use reporting in which Thais under the age of 19 were found to use the Internet *12.8 h a day* (Manakitsomboom, 2021), while Thais 19–38 years old spent *12.26 h a day* online.

### 5.2 Student opinions of online learning by gender

Additional analysis of the Thai online education process used by extra-large high schools at the time of the survey showed that female students had a slightly higher willingness to continue the online lessons (student retention) as the mean = 3.01 and the SD = .58. When course quality was evaluated, the numbers were nearly reversed as males thought it was slightly better (mean = 3.01, SD = .55) than their female classmates (mean = 2.98, SD = .60). Also, online learning satisfaction was viewed almost equally by both male (mean = 2.98, SD = .48) and female (mean = 3.00, SD = .52) students. Finally, as we can see from all aspects in Table 9, there is room for improvement as the students had an overall *moderate* opinion concerning their online educational experiences. This is consistent with other current reports and studies (Imsa-ard, 2021; Wangkiat, 2021).

**Table 9:** Student opinions of online learning by gender.

Gender	Student retention			Course quality			Total satisfaction		
	Mean	SD.	Level	Mean	SD.	Level	Mean	SD.	Level
Male (n = 102)	2.97	.56	moderate	3.01	.55	moderate	2.99	.48	moderate
Female (n = 168)	3.01	.58	moderate	2.98	.60	moderate	3.00	.52	moderate
Total (n = 270)	3.00	.57	moderate	3.00	.58	moderate	3.00	.51	moderate

### 5.3 Student opinions of online learning by region

In Table 10, additional analysis of the Thai online education process being used by extra-large high schools at the time of the survey showed that students from the southern province of Trang were significantly more satisfied with their online learning experiences when compared to students from other Thai regions in student retention (mean = 3.30, SD = .52), course quality (mean = 3.27, SD = .52), and total satisfaction (mean = 3.28, SD = .52). However, across all nine provinces survey in their four regions, online learning student retention, course quality, and total satisfaction were judged to be at a ‘*moderate*’ level.

**Table 10:** Student opinions of online learning by region.

Region	Student retention			Course quality			Total satisfaction		
	Mean	SD.	Level	Mean	SD.	Level	Mean	SD.	Level
Northern (n = 30)	3.03	.60	moderate	2.87	.57	moderate	2.93	.49	moderate
Central (n = 120)	2.98	.55	moderate	3.01	.53	moderate	3.00	.47	moderate
Northeast (n = 90)	2.90	.48	moderate	2.92	.55	moderate	2.91	.46	moderate
South (n = 30)	3.30	.77	moderate	3.27	.77	moderate	3.28	.71	moderate
Total (n = 270)	3.00	.57	moderate	3.00	.58	moderate	3.00	.51	moderate

### 5.4 Performance expectancy (PE)

First, it was determined that H1 was unsupported (*performance exp*ectancy - > *behavioral intention*). However, H2 was supported with the relationship from PE - > SS being moderately strong (*r* = 0.47, t-value = 3.20, *p* ≤ 0.01). Furthermore, we note the importance the online students placed on perceived benefit (x3 = 0.89) and their intrinsic motivation (x1 = 0.80), which is supported by a study from Fagan et al. (2008) in which the authors revealed that there was a positive relationship between *intrinsic motivation* and *perceived ease of use* and a positive relationship between *perceived ease of use* and BI to use computers. These results are also consistent with Teo et al. (2019), whose investigation into university student use of Moodle online in Macau stated that ease of use and usefulness were significant from the sample of students’ attitudes towards using Moodle. Moodle also played a predominant role in online education research from Ghana, in which Yawson and Yamoah (2020) stated that open-sourced learning management networks such as Moodle had gained global appeal and acceptance in higher education. Finally, in Greece, Krouska et al. (2021) determined that the *perceived ease of use* and *enjoyment* significantly affect mobile game-based learning (MGBL).

### 5.5 Effort expectancy (EE)

Results also determined that there was weak but positive relationship from *effort expectancy* - > *actual use* as *r* = 0.25, t-value = 2.20, *p* ≤ 0.05. This is consistent with other studies from our research in which course design (x5) is crucial to *student satisfaction* and a student's *actual us* (Lee, 2014; Liu et al., 2010; Sun and Chen, 2016).

Thus, students will utilize these channels to engage in their class and create an interactive learning environment if interactive features such as a discussion room and chat room are introduced to an online course. Furthermore, live streaming collaboration technologies such as Zoom, Microsoft Team, and Google Meet make it simpler for students to communicate effectively with their peers and professors than ever before (Kanetaki et al., 2021).

However, as Woodworth et al. (2015), a common reason for student dissatisfaction with online learning is poor course design, lacking supervision, and poor pedagogy in online instruction. Thus, these factors can contribute to poor learning outcomes and low enthusiasm for the online learning format. Also, in Greece, Kanetaki et al. (2021) showed that when online CAD modules were used, outcomes were more pleasurable when implemented through a learning management system such as MS Teams. Finally, the intention to use online learning AR systems is positively influenced directly by system quality and perceived ease of use (Papakostas et al., 2021).

### 5.6 Social influence (SI)

The relationship from *social influence* - > *behavioral intention* in H4 was also weakly but positively supported as *r* = 0.28, t-value = 2.03, *p* ≤ 0.05. This is consistent with Venkatesh (2000), which determined that although attitude was more important for men, subjective norms and perceived behavioral control were essential for women in the early stages of technological adoption. Also, in Turkey, Baki et al. (2021) examined distant learning on student intention to use and reported that self-efficacy, subjective norms, and enjoyment did not influence the 925 students in the sample.

### 5.7 Learner interaction (LI)

In the relationship in H5 from *learner interaction* - > *behavioral intention*, we determined it was moderately strong in its support. This was due to *r* = 0.47, t-value = 2.63, *p* ≤ 0.01. However, the relationship from *learner interaction* - > *student satisfaction* in H6 was not supported.

Results from H5 are consistent with both Asmuni et al. (2012) and Pimdee and Leekitchwatana (2022), which in their studies reported that students observe their surrounding classmates from the use of social media, peer pressure, their family's interaction.

Moreover, it was determined that LI did not directly affect SS due to deficiencies in the methods and systems being used. As we have learned from other studies, LMS platforms such as Moodle contribute significantly to online learning satisfaction and collaboration. However, we observed that many courses are being conducted online without the aid and assistance of a *learning management system*. Therefore, *learner interaction* with their classmates, teachers, and assignments breaks down (Kanetaki et al., 2021; Krouska et al., 2021; Teo et al., 2019; Yawson and Yamoah, 2020).

### 5.8 Facilitating conditions (FC)

In the study's examination of *facilitating conditions*, two hypotheses were conceptualized. The first one was H7, in which the relationship from *facilitating conditions* - > *behavioral intention* was found to be supported but weak. This was due to *r* = 0.21, t-value = 2.40, *p* ≤ 0.05. In the second hypothesis for *facilitating conditions* (H8), the relationship from *facilitating conditions* - > *student satisfaction* was determined to be unsupported.

The lack of support in H8 was speculated to be due to the requirements that the Thai government has to provide Internet connectivity and supporting infrastructure (Chardnarumarn et al., 2021; Oranop, 2016; Ruenphongphun et al., 2021). Thus, FC does not affect SS.

Furthermore, the two latent variables, *learner interaction* and *facilitating conditions* (FC), had no direct or indirect influence on *student satisfaction*, unlike what many scholars had previously found to be true. However, this study's findings are in line with the context of the learning management situation during the Covid-19 period. Therefore, students are now limited with regards to their LI and FC.

These results find support in a study conducted on university students in both Indonesian (352) and Thailand (380), in which the authors stated that in online courses in Thailand, *service quality* (SQ) reliability was judged to be most important (Darawong and Widayati, 2021). Moreover, in Thailand, the strongest dimension of *service quality* affecting *student satisfaction* was reliability, responsiveness, and competence, respectively. However, the Indonesian university students' most substantial *student satisfaction* effect on *service quality* was empathy, responsiveness, competence, and reliability.

Moreover, diving deeper, we see that the observed variables *ICT infrastructure* (x11) and *Internet availability* (x12) are perceived by other studies’ online students as crucial elements to SS (Bisht et al., 2020; Mendoza et al., 2017; Nonthamand et al., 2021).

### 5.9 Behavioral intention (BI)

In hypothesis H9, the relationship from *behavioral intention* - > *actual use* was found to be moderately strong and supported due to *r* = 0.41, t-value = 3.87, *p* ≤ 0.01. This result is consistent with research from South Korea in which Park et al. (2012) investigated higher education mobile learning (m-learning) and reported that the student's *attitude* was most important, followed by their *study discipline* and *subjective norm*.

### 5.10 Actual use (AU)

Finally, in the study's tenth and final hypothesis, H10, the relationship from *actual use* - > *student satisfaction* was found to be moderately strong and supported due to *r* = 0.30, t-value = 3.56, *p* ≤ 0.01. This finding is supported by research from Thongsri et al. (2019), which determined that online course *performance expectancy, social influence, information quality*, and *system quality* significantly affect *intention to use*. These findings are also consistent with research in Greece concerning artificial reality (AR) in firefighter training. Papakostas et al. (2021) determined that usability is the strongest predictor of a trainees' behavioral intentions to use the AR system.

Also, *student satisfaction* has been influenced by *actual use* (Aldholay et al., 2018; Hou, 2012; Isaac et al., 2017). However, there were no significant correlations between EE, *social influence*, *learner interaction*, and *facilitating conditions*. Even so, there were still interactions between those factors. This might be because, during the pandemic, students consider that studying online is a requirement. As a result, the relationship between *behavioral intention* and *actual use* might be unusual, and since they were mediators between other exogenous latent factors and student satisfaction, the degree of effect was not as substantial as predicted.

### 5.11 Concerns and follow-up comments

The results raise a worrying concern about whether all Thai schools are held to the same educational standards and whether entrance exams assess students' knowledge outside the curriculum. Also, although other studies are reporting that Thais younger than 39 years of age are using the Internet and online social media over 12 h a day, in the opened questionnaire response, most revealed that studying online for more than 6 h a day was excessive.

Another essential aspect to note is that although the smartphone was the most commonly used digital device, we observed that smartphones have a small screen, thus making it unsuitable (in our opinion) for taking courses longer than an hour. However, it is good to report that most of the students at the time of the survey already had a digital device (89.60%) to connect with their online course and a Wi-Fi signal (68.90%), although the average bandwidth and throughput was undetermined.

As can be seen, students in Thailand now have access to ICT and Internet infrastructure; however, the robustness of the connections and underlying technology has yet to be established. As mentioned previously, Thai students recently took to the streets in Thailand's capital Bangkok to express their anger concerning their online education satisfaction (Wangkiat, 2021), part of which comes from the additional costs being absorbed by students and their families for online learning and Internet access time/data use.

In response to the open-ended question, 22.22% of the student respondents offered an opinion, with most voicing concerns about the design of the online courses, including their perception of the excessive nature of excessive homework. Also, some students felt that the online learning exam method was inapplicable and unsuitable for most students. Some also claimed that some of their teachers' online teaching methods were a waste of time and that their schools’ online learning systems were chaotic and not well-organized.

Moreover, students acknowledged that the length of time spent online learning was important, not only for the efficiency of their learning process but also because hour-long sessions had a negative impact on their physical health. There is support for this complaint as the *Thailand Physical Activity Knowledge Development Centre* (TPAK) has backed up this claim (Diawkee, 2021; Katewongsa et al., 2021). Students also said that teachers might be crucial in making students satisfied with online learning, but only a small percentage of them could do this.

Furthermore, all expected observed factors for *performance expectancy, effort expectancy*, *social influence*, *learner interaction*, and *facilitating conditions* could explain the influence of those observed variables. However, only plan to use (y2), usage time (y4), and course quality (x6) were found to be able to explain *behavioral intention*, *actual use*, and SS, respectively. This was speculated as being influenced by the present conditions of mandatory online learning in Thailand, with the satisfaction score not reflecting a high level of *student satisfaction*. Thus, this may result in low student attendance and retention rates.

Finally, the study confirmed that the model adequately explained causal relationships between variables and presented direct and indirect significant impacts on online student satisfaction. This then can promote a learner's better academic performance and knowledge acquisition (Troussas et al., 2021).

## 6 Conclusion

We set out to examine the current state of online learning in Thailand how it affects Thai secondary school student satisfaction. Results revealed that during the COVID-19 pandemic, *performance expectancy* and *actual use* was the predictor of online learning *student satisfaction* (Sig. ≤ .05). Furthermore, *student satisfaction* may be predicted by the degree of *learner interaction* and *facilitating conditions* (not significant).

Course design from *effort expectancy* factors and all three forms of interaction were also addressed in the answers to the open-ended question on enhancing Thailand's online learning systems. Furthermore, since many replies indicated political or vaccination-linked answers, several students still have an emotional bias from the country's political concerns. Moreover, it is argued that the pandemic's negative sentiments may have influenced students' attitudes toward online learning (Aguilera-Hermida, 2020).

Overall, the surveys led us to believe that online education in Thailand is unsuccessful, with SS scores with online learning not exceptionally high. Therefore, it may be concluded that students in Thailand are dissatisfied with the current online learning system, which is being reflected in the daily media, other concurrent studies, and reports. There are still many things that need improvement; therefore, the government or other stakeholders might initially focus on improving aspects connected to *performance expectancy* to boost *student satisfaction*.

This research also highlights the plight of high school students with online learning during the COVID-19 pandemic in Southeast Asia. As educators and administrators rushed to solve the lockout and distancing dilemmas in continuing the education and learning process (Kanetaki et al., 2021; Krouska et al., 2021), very little attention or focus was given to how ‘online learning’ would affect the millions of students who were now forced to use it. As can be seen from Thailand's ‘bad student’ movement (Wangkiat, 2021), Thai students ‘*dissatisfaction’* with online learning has become headlines across many global publications and even entered very recent academic studies (Imsa-ard, 2021). With most research on this idea coming from western and developed economies, few 'real-time' studies have been undertaken yet while these events are still unfolding in Southeast Asia.

However, this study and its authors quickly identified the problem under extreme and limiting conditions undertook a survey across a broad geographic spectrum within Thailand with a follow-on SEM study involving eight latent variables and ten hypotheses. We, therefore, feel that this study is highly original in its scope, very timely in its execution, unique when compared to other studies attempting to cover the same topic under the same conditions, and is one of the first to detail what factors affect a student's online course satisfaction and propose solutions.

## 7 Potential study limitations and future suggestions

Although the study took place in 2021 under severe lockdown conditions precipitated by the multi-year COVID-19 pandemic, we managed to organize a survey of 270 students using local teacher assistance on online questionnaire response using Google Form. However, we believe that follow-on studies under less constraining conditions can reveal greater detail from a more comprehensive sampling group, possibly ASEAN (Association of Southeast Asian Nations) in nature.

Also, this research collected data from students in extra-large schools. The findings may not reflect students' views from small and medium-sized schools in Thailand, other ASEAN nations, or more developed nations. We want this research to be treated as a case study of Thailand's online education system. We do not want to say that this result will be the same in other countries, as different countries have different ways to manage their online education system. Also, students from collectivist countries and individualist countries may have different results.

Because this study has a limitation that may impact the results, we cannot dismiss the non-significant effects of other latent factors. There also needs to be better determinations concerning just how much bandwidth online students are receiving from their primary point of connection to their online courses, how much it costs, and the carrier used to provide the link.

## 8 Contribution to the research

This study was conducted deep into the ongoing global COVID-19 pandemic emergency. Despite this, under very constraining conditions, we managed to survey 270 students who were involved daily with taking courses online within Thailand's highly controversial online education system. As such, the study is one of the first to detail what factors affect a student's online course satisfaction and propose solutions. This study's research contribution is that it is also unique in that it was conducted during the pandemic lockdown while students were participating in Thai Ministry of Education (MOE) online courses. The results also have significant practical implications for educational institutions and decision-makers regarding course design, online systems, and student retention. We feel it is a study with a high level of interest globally, from course instructors to cabinet-level ministers.

## Declarations

### Author contribution statement

Sudaporn Sawmong: Conceived and designed the experiments; Analyzed and interpreted the data.

Piriyakorn Kornpitack: Conceived and designed the experiments; Performed the experiments; Analyzed and interpreted the data; Contributed reagents, materials, analysis tools or data; Wrote the paper.

### Funding statement

This research did not receive any specific grant from funding agencies in the public, commercial, or not-for-profit sectors.

### Data availability statement

Data included in article/supplementary material/referenced in article.

### Declaration of interests statement

The authors declare no conflict of interest.

### Additional information

No additional information is available for this paper.
